# FGT-1 is the major glucose transporter in *C. elegans* and is central to aging pathways

**DOI:** 10.1042/BJ20131101

**Published:** 2013-11-08

**Authors:** Ying Feng, Barnabas G. Williams, Françoise Koumanov, Adrian J. Wolstenholme, Geoffrey D. Holman

**Affiliations:** *Department of Biology and Biochemistry, University of Bath, Bath, BA2 7AY, U.K.; †Department of Infectious Diseases, University of Georgia, Athens, GA 30602, U.S.A.

**Keywords:** aging, *Caenorhabditis elegans*, facilitated glucose transporter, isoform 1 (fgt-1), glucose transport, insulin, metabolism, AGE-1, ageing alteration 1, AMPK, AMP-activated protein kinase, DAF, abnormal dauer formation, 2DG, 2-deoxy-D-glucose, FUDR, 5-fluoro-2′-deoxyuridine, GLUT, glucose transporter, HA, haemagglutinin, hGLUT1, human GLUT1, IIS, insulin/insulin-like growth factor-like signalling, KRH, Krebs–Ringer Hepes, NGM, nematode growth medium, PI3K, phosphoinositide 3-kinase, qPCR, quantitative real-time PCR, ROS, reactive oxygen species, RT-PCR, reverse transcription–PCR, TM, transmembrane

## Abstract

*Caenorhabditis elegans* is widely used as a model for investigation of the relationships between aging, nutrient restriction and signalling via the DAF-2 (abnormal dauer formation 2) receptor for insulin-like peptides and AGE-1 [ageing alteration 1; orthologue of PI3K (phosphoinositide 3-kinase)], but the identity of the glucose transporters that may link these processes is unknown. We unexpectedly find that of the eight putative GLUT (glucose transporter)-like genes only the two splice variants of one gene have a glucose transport function in an oocyte expression system. We have named this gene *fgt-1* (facilitated glucose transporter, isoform 1). We show that knockdown of *fgt-1* RNA leads to loss of glucose transport and reduced glucose metabolism in wild-type worms. The FGT-1 glucose transporters of *C. elegans* thus play a key role in glucose energy supply to *C. elegans.* Importantly, knockdown of *fgt-1* leads to an extension of lifespan equivalent, but not additive, to that observed in *daf-2* and *age-1* mutant worms. The results of the present study are consistent with DAF-2 and AGE-1 signalling stimulating glucose transport in *C. elegans* and this process being associated with the longevity phenotype in *daf-2* and *age-1* mutant worms. We propose that *fgt-1* constitutes a common axis for the lifespan extending effects of nutrient restriction and reduced insulin-like peptide signalling.

## Abstract 2

Only the FGT1A/B protein isoforms function as glucose transporters in *C. elegans*. Knockdown of fgt-1 RNA leads to loss of glucose transport, reduced glucose metabolism and extended lifespan. FGT-1 glucose transporters therefore play a key role in regulated glucose energy supply and lifespan.

## INTRODUCTION

Endocrine regulation of glucose uptake is widely studied in mammals and particularly humans with an emphasis on insulin sensitivity and insulin resistance. In mammals a family of glucose transporter proteins (the GLUT family) is primarily responsible for maintaining circulating glucose levels. The regulation of the GLUT4 protein of insulin-target tissues is well established as occurring downstream of the insulin receptor and PI3K (phosphoinositide 3-kinase) signalling [1] and is known to become dysfunctional in human obesity and Type 2 diabetes [2–4]. As emphasized by Ristow and colleagues, treatments for obesity and diabetes with associated insulin resistance are based on the idea that increasing insulin sensitive glucose uptake, with a consequent lowering of blood glucose, will improve health outcomes. However, studies on knockout of signalling by receptors for insulin or insulin-like peptides [IIS (insulin/IGF (insulin-like growth factor)-like signalling] indicate that IIS may have adverse consequences on healthy aging [5–7]. Healthy aging is likely to be dependent on maintaining the expression and functional activities of endocrine-regulated nutrient transporter proteins. These processes are difficult to study in mammals and humans because of their long lifespans. By contrast, *Caenorhabditis elegans* is widely used as a model organism for studies linking nutrient uptake and cell signalling via insulin-like peptides to aging, because of the much shorter lifespan of this organism.

Supplying high glucose levels to *C. elegans* leads to a marked reduction in their lifespan which is mediated by down-regulation of DAF-16 (abnormal dauer formation 16) with consequent effects on gene transcription [8]. Conversely, nutrient restriction leads to an extension of lifespan [6,9]. A range of mechanisms have been proposed to account for the effects of nutrient restriction on lifespan [10]. Most recently emphasis has been placed on the role played by increased mitochondrial respiratory activity. Increased mitochondrial activity leads in turn to ROS (reactive oxygen species) signals and induction of a stress response programme that facilitates longevity [11]. An important intermediate in this pathway in *C. elegans* is AAK-2/AMPK (AMP-activated protein kinase) which is activated in DAF-2- and glucose-uptake-deficient worms [7].

Endocrine control of lifespan is well established in *C. elegans* with mutations in the genes encoding the receptor for insulin-like peptides, DAF-2 [12,13] and AGE-1 (ageing alteration 1)/PI3K [14,15] leading to extended lifespan. Signalling via the DAF-2 receptor and AGE-1/PI3K are thought to lead to phosphorylation of DAF-16 and block of its transcriptional activity. Reduction in this signalling therefore leads to increased expression of longevity genes and decreased expression of pro-aging genes [10]. The possibility that in *C. elegans* a parallel pathway might occur from the DAF-2 receptor and AGE-1/PI3K leading to glucose transport, similar to that occurring in mammals [4], has been previously hypothesized [7], but the identity of the glucose transporter that is involved has not been determined.

Currently very little is known of the process by which nematodes take up glucose. In the present study we address this problem by characterizing the GLUT-like proteins in *C. elegans* and by investigating the relationship of these proteins with glucose- and signalling-dependent aging. During this search for *C. elegans* glucose transporters we unexpectedly discovered that very few of the GLUT-like gene sequences described in WormBase are likely to code for functional glucose transporters.

## EXPERIMENTAL

### *C. elegans* strains and their maintenance

Worms were cultured and maintained as described previously [16]. The following strains were used: wild-type (N2), CB 1370 *daf-2*(*e1370*) and TJ 1052 *age-1*(*hx546*). Mutant strains were all provided by the Caenorhabditis Genetics Center (University of Minnesota, Minneapolis, MN, U.S.A.).

### Protein sequences alignment

The membrane topology of the candidate putative transporters was predicted using the SOSUI program [17]. The protein sequence of each protein was also aligned with hGLUT1 (human GLUT1) using the T-Coffee program [18]. The TM (transmembrane) segment assignments of the putative transporters were set up to maximize alignment with the hGLUT1 TM regions [19], the XylE transporter of known 3D structure [20] and at the same time maintain sufficient residues in the assigned TM region predicted by SOSUI.

### Cloning of candidate glucose transporter genes and HA (haemagglutinin) insertion into the first exofacial loop of the target genes

Total RNAs of mixed stage N2 worms were isolated with the SV Total RNA Isolation System (Promega). First-strand cDNA was synthesized using SuperScript™ III First-Strand Synthesis for RT-PCR (reverse transcription–PCR) (Invitrogen). The candidate genes were amplified from the cDNA library using primers which were designed according to the predicted spliced forms of the gene sequence in the WormBase (http://www.wormbase.org/). Forward and reverse primers for amplifying the target genes are listed in Supplementary Table S1 (at http://www.biochemj.org/bj/456/bj4560219add.htm). The amplified genes were first cloned into pT7Blue vector for subsequent manipulation using Perfectly Blunt® Cloning Kits (Novagen). The influenza HA epitope was inserted into the predicted first exofacial loop of the target proteins as described previously [21] and introducing a Bsu36I restriction site as indicated in Supplementary Table S2 (at http://www.biochemj.org/bj/456/bj4560219add.htm). The peptide sequence was IDYPYDVPDYAE, the sense oligonucleotide was 5′-TGAGATCGATTATCCTTATGATGTTCCTGATTATGC-3′ and the antisense oligonucleotide was 5′-TCAGCATAATCAGGAACATCATAAGGATAATCGATC-3′. The sequence of each construct was analysed by the sequencing service provided by GATC Biotech.

### Heterologous expression of putative glucose transporters in the *Xenopus* oocytes

The putative glucose transporter genes (with or without a HA tag) were cloned into *Xenopus laevis* expression vector pT7TS (Addgene plasmid 17091 provided by Professor Paul Krieg, University of Arizona, Tucson, AZ, U.S.A.) which was flanked by fragments of the 5′- and 3′-untranslated regions of *Xenopus* β-globin mRNA. The constructs were linearized at the 3′-end of the putative transporter genes. cRNAs were synthesized using the T7 RNA polymerase in the presence of cap analogue (mMESSAGE mMACHINE, Ambion) and purified using phenol-chloroform extraction. Stage V or VI oocytes were isolated from *X. laevis* females by digestion of ovarian lobes with 1.5 mg/ml collagenase type II (Sigma) in Ca^2+^-free OR-2 buffer [82.5 mM NaCl, 2.5 mM KCl, 1 mM Na_2_HPO_4_, 1 mM MgCl_2_ and 5 mM Hepes (pH 7.5)] at 15°C for 1.5 h. After an overnight incubation in ND96 buffer [96 mM NaCl, 2 mM KCl, 1 mM MgCl_2_, 1.8 mM CaCl_2_, 5 mM Hepes (pH 7.5), 2.5 mM pyruvic acid and 1% FBS], healthy oocytes were selected for injection with 50 nl (50 ng) of capped RNA coding for the putative glucose transporters or with the same volume (50 nl) of DEPC (diethyl pyrocarbonate)-treated water. To express the transporters, the oocytes were incubated in ND96 buffer at 18°C for 3 days with the medium changed every day.

### Surface detection of HA-tagged transporters in *Xenopu*s oocytes

A total of five oocytes expressing the HA-tagged transporters were treated in 100 μl of KRH (Krebs–Ringer Hepes) buffer with an anti-HA antibody (1 μg/ml for 2 h at 25°C). After three washes in KRH buffer the cells were incubated for a further 1 h at 25°C in presence of 1 μg/ml anti-(mouse IgG–β-galactosidase conjugate) (Southern Biotech). Cells were then transferred on to 96 well plates and the fluorogenic β-galactosidase substrate fluorescein-digalactoside (Invitrogen) was added to give a final concentration of 0.1 mM. The resulting fluorescence was measured every 15 s over a 60 min period in a Pherastar FS multiwell plate reader (BMG). The level of HA-tagged transporters present at the cell surface was then calculated from the rate of fluorescence generated per oocyte.

### Glucose transport activity in *Xenopus* oocytes

Healthy *Xenopus* oocytes injected with cRNA or H_2_O were washed with incomplete ND96 buffer. The glucose-transport activity of the oocytes was determined by incubating groups of five oocytes in 200 μl of incomplete ND96 buffer with the indicated final concentrations of D-glucose for 30 s before the addition of [U-^14^C]glucose (Amersham, GE Healthcare; 0.3 μCi per assay). The reaction was stopped after 60 min by rapidly washing the oocytes three times with ice-cold incomplete ND96 buffer containing 300 μM phloretin. Each group of oocytes was solubilized with 1% (w/v) SDS and the radioactivity present was determined by liquid scintillation counting.

### RNAi constructs and RNAi feeding

The *fgt-1a* and *fgt-1b* RNAi constructs were obtained by subcloning a 417 bp DNA fragment targeted to exon 1 of *fgt-1a* and a 395 bp DNA fragment targeted to exon 2 of *fgt-1b* from the pT7Blue vector into the L4440 vector. After transformation with either the L4440-*fgt-1a* or -*fgt-1b* RNAi construct, HT115 strain bacteria were grown in LB medium with ampicillin (50 μg/ml) for 8 h, then seeded on to NGM (nematode growth medium) plates containing ampicillin (50 μg/ml) and IPTG (1 mM) and then induced overnight. Synchronized and starved L1 larvae were seeded on to the NGM plates with a RNAi bacteria food source. The worms were allowed to grow on the RNAi plates without exhausting the bacteria food and until the young adult stage. The extent of knockdown of total *fgt-1a* and *fgt-1b* mRNA was determined by qPCR (quantitative real-time PCR). Total RNAs from the worms were isolated with the SV Total RNA Isolation System (Promega). cDNAs were synthesized with random hexamers using SuperScript™ III First-Strand Synthesis for RT-PCR system (Invitrogen). PCR was performed using iTaqTM SYBR® Green Supermix with ROX kit in a real-time thermal cycler (Mx3000P QPCR system, Stratagene). The PCR primers used for *fgt-1a* and *fgt1b* were: forward, 5′-GGCCAGCTACTCAGCCATC-3′ and reverse, 5′- ATTTCGGAGACGAAGAACCA-3′. The gene *act-1* (forward primer, 5′-CCAGGAATTGCTGATCGTATGCAGAA-3′ and reverse primer, 5′-TGGAGAGGGAAGCGAGGATAGA-3′) was used as housekeeping gene to normalize the RNA level of *fgt-1*.

### 2DG (2-deoxy-D-glucose) transport activity of RNAi-treated worms

Synchronized L1 worms were grown on NGM plates (50 μg/ml ampicillin) with or without 1 mM IPTG to induce RNAi expression seeded with appropriate bacteria until the young adult stage. The worms were then transferred on to NGM plates (50 μg/ml ampicillin) containing 50 μM FUDR (5-fluoro-2′-deoxyuridine) and seeded with appropriated bacteria with or without 1 mM IPTG. The adult worms were allowed to grow on FUDR NGM plates for 10 days without exhausting the bacteria food source. On the day of the experiment, worms were washed with M9 buffer (22 mM KH_2_PO_4_, 22 mM Na_2_HPO_4_, 85 mM NaCl and 1 mM MgSO_4_) to completely remove the bacteria. The worms, in 500 μl of M9 buffer, were incubated for 2 h with 500 μM 2-DG (with 1 μCi of [^3^H]2DG per assay). The worms were then pelleted and rapidly washed with ice-cold M9 buffer four times. The mixture was sonicated at amplitude of 27 microns five times for 15 s with intervals of 60 s on ice. The suspension was then centrifuged at 14000 ***g*** at 4°C for 15 min. A 50 μl aliquot of the supernatant was saved for a protein assay. The radioactivity in the worm lysates was determined using a scintillation counter.

### Glucose oxidation and incorporation into lipid of RNAi-treated worms

Worms were prepared as described in the 2DG-transport assay. Uniformly labelled [^14^C]D-glucose oxidation was determined as described previously [6] with some modification. Each sample was analysed in triplicate. Worm pellets were added to 5 ml Bijou tubes. An open 1.5 ml tube containing 1 ml of 0.1 M KOH was placed into the Bijou tube to trap the CO_2_ which was produced. The worms, in 500 μl of M9 buffer, were incubated for 3 h at 18°C with 500 μM D-glucose in M9 buffer (with 1 μCi of [U-^14^C]D-glucose per assay). The uptake was stopped by flash-freezing the worm suspension in liquid nitrogen. The radioactivity trapped in the KOH solution was determined using a scintillation counter. The frozen worms were then thawed. The mixture was sonicated on ice with a tip sonicator. The suspension was then centrifuged at 14000 ***g*** at 4°C for 15 min. A 50 μl aliquot of the supernatant was saved for a protein assay. The fat content was extracted from the supernatant by the addition of heptane. The radioactivity in the organic phase was determined using a scintillation counter.

### *C. elegans* lifespan

Lifespan assays were performed at 20°C. Young adult worms were allowed to lay eggs overnight on NGM plates (50 μg/ml ampicillin) with or without 1 mM IPTG to induce RNAi expression and seeded with RNAi bacteria. The progeny were allowed to grow on the corresponding plates until the L4 larvae stage at 18°C. The L4 larvae were then transferred on to NGM plates (50 μg/ml ampicillin) containing 50 μM FUDR seeded with appropriate bacteria with or without 1 mM IPTG and maintained at 20°C without the exhaustion of the bacteria food and until all the worms on the plate had died. The adult population was scored every day or every other day. Animals that crawled off the plates or exploded were censored. Animals that failed to respond to a gentle prodding with a platinum wire were scored as dead. Day 0 of the lifespan was set as the day that the eggs were laid.

### Statistical analysis

All analyses were performed using GraphPad Prism version 5.01. *C. elegans* survival curves were analysed using the Kaplan–Meier method. Unpaired two-tailed Student's *t* tests were carried out using a 95% confidence interval. Differences were considered to be significant at *P* values of <0.05.

## RESULTS

### Characterization of *C. elegans* GLUT-like genes

**Figure 1:**
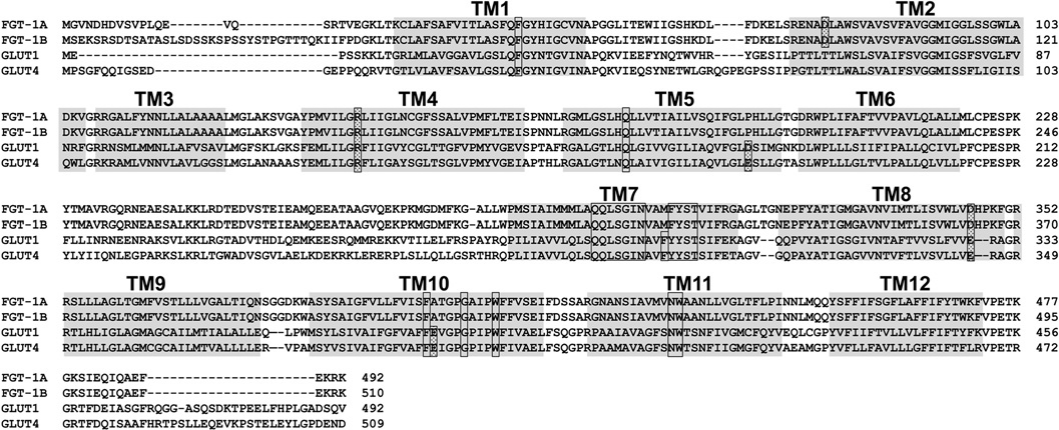
Comparison of FGT-1A and -1B with GLUT1 and GLUT4 Alignment allows comparison of invariant and non-charged residues, particularly in the C-terminal half of the protein. Boxes with a dotted-fill highlight charged residues in the TM regions. Highly conserved signature residues (boxed residues), particularly in the TM7 region, are thought to be important in substrate recognition (see the Supplementary Online Data at http://www.biochemj.org/bj/456/bj4560219add.htm for more information).

Eight gene sequences with significant homology to members of the mammalian GLUT family of glucose transporters have been identified in the *C. elegans* genome sequence (Table 1). These putative glucose transporter genes all code for proteins (Figure 1 and Supplementary Figure S1 at http://www.biochemj.org/bj/456/bj4560219add.htm) that have signature motifs related to the general MFS (major facilitator superfamily) structural architecture and to the mammalian GLUT family of proteins [20,22]. This is likely to be why bioinformatics approaches identify this large number of *C. elegans* sequences as belonging to the sugar transporter family.

**Table 1:** Putative glucose transporter (GT) proteins which have sequence homology with mammalian glucose transporters of the GLUT family WormBase has assigned putative GLUT-like transporters genes in *C.elegans* to orthologues in the human GLUT family. Although the WormBase identities are relatively high, these assignments do not necessarily predict the preferred substrate for the transporter proteins such as D-glucose. H17B01.1 (which we have now been reassigned the gene name *fgt-1*) has a high identity with GLUT3, GLUT1 and GLUT4. This gene codes for two splice variants. WormBase-listed RNAi phenotyping of some of the expressed genes has led to the indicated traits. No phenotype associated with RNAi phenotyping of *fgt*, *R09B5.11* or *K09C4.1* has been described in WormBase.

Putative GT (gene name)	Amino acids	Assigned human orthologue in WormBase	Identity with assigned orthologue	Identity with hGLUT1	Identity with hGLUT4	RNAi phenotype
H17B01.1 A	492	GLUT3	43%	37%	36%	
H17B01.1 B	510	GLUT3	43%	37%	36%	
R09B5.11	516	GLUT1	36%	36%	33%	
C35A11.4	515	GLUT3	29%	26%	25%	Larval lethal, embryonic lethal
F14E5.1	472	GLUT14	27%	24%	26%	Fat content increase
Y39E4B.5	505	GLUT9	24%	20%	23%	Larval rest
F48E3.2	488	GLUT1	23%	23%	23%	Embryonic lethal
K09C4.5	524	GLUT14	22%	18%	24%	Fat content increase
K09C4.1	521	GLUT4	20%	17%	20%	

**Figure 2:**
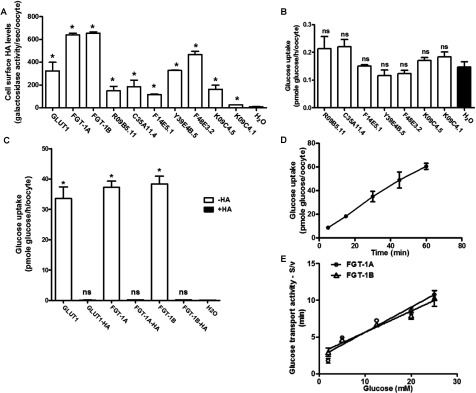
Glucose transport activity of *C. elegans* GLUT-like transporters expressed in *Xenopus* oocytes (**A**) The levels of GLUT-like transporters at the cell surface as determined using exofacial HA epitopes. cRNAs for transporters HA-tagged in the first exofacial loop were injected into oocytes. The HA tag at the cell surface was detected with an anti-HA antibody and a β-galactosidase-conjugated secondary antibody. Results are means±S.E.M. for three separate experiments. *P<0.05 compared with the water-injected control oocytes. (**B**) Glucose-transport activity associated with the indicated putative GLUT-like transporters (without HA tags). Results are means±S.E.M. for three separate experiments. ns, not significantly different compared with the water-injected control oocytes. (**C**) Comparison of glucose-transport activities, in the presence and absence of the HA tags, that are associated with the expression of hGLUT1 and *C. elegans* FGT-1A and FGT-1B. Results are means±S.E.M. for three separate experiments. *P<0.05 compared with the water-injected control oocytes. (**D**) Uptake of 50 μM D-glucose via FGT-1A expressed in oocytes. Time courses were found to be linear. Results are means±S.E.M. for three separate experiments. (**E**) Rates of maximal transport for glucose in *Xenopus* oocytes expressing 50 ng of cRNA for FGT-1A and FGT-1B. Oocytes were incubated with the indicated concentrations of D-glucose (S). The rates of [^14^C]glucose uptake (v, pmol glucose/oocyte per min) were used in least-square fits to the Michaelis–Menten equation. Results are means±S.E.M. for three separate experiments.

We examined the functional activity of the putative *C. elegans* glucose transporters by expressing them in *Xenopus* oocytes. As functional activity is dependent upon the extent to which the transporters are incorporated into the plasma membrane we initially introduced the influenza HA epitope tags into the first exofacial loop of each of the transporters. This approach has been used extensively for studies of the function of the mammalian transporter GLUT4 [23]. Using this approach we found that all the transporters were significantly, but variably, expressed at the cell surface (Figure 2A); however, it became apparent that introduction of the HA tag led to a complete loss of glucose transport activity for all the transporters, including mammalian GLUT1, that we examined in the oocyte expression system (Figure 2C). The level of reduction in transport activity was not trivial. In the case of GLUT1, transport was reduced by over 99% and a level that was not significantly different from the water injection control. As introduction of HA epitope tags was not a viable option for comparing the putative *C. elegans* glucose transporters we chose to expresses these transporters without the tag. The disadvantage here is that the cell-surface expression level is unknown. However, the studies using the HA tag suggest that although traffic to the cell surface is variable, it is always present to some extent and we therefore assumed that the same will apply to the non-tagged constructs. Using this approach we found that the only proteins that significantly transported glucose were the two products of the gene H17B01.1. Even allowing for variations in levels of surface availability of the transporters it is evident that only these gene products can clearly function as glucose transporters. We have therefore re-named the H17B01.1 gene as *fgt-1* and the transporter proteins as FGT-1A and FGT-1B (Figures 2B and 2C). These proteins transport glucose at levels that are comparable with GLUT1 expressed in the same system and at rates that are approximately 200 times that of R09B5.11, the most comparable homologue amongst the other transporters examined, and the water-injection control.

Although comparison of the FGT-1A and FGT-1B sequences reveals that the latter has an N-terminal 18 amino acid extension, both the HA-tagged forms of these transporters are well expressed at the cell surface of oocytes (Figure 2A) and have similar affinity (*K*_m_≈7–9 mM) and transport catalytic activity (*V*_max_≈3 pmole/min per oocyte) at the comparable (50 ng) levels of cRNA that were injected (Figures 2D and 2E). The functional consequences of the N-terminal portion are therefore unrelated to catalytic activity and some regulatory role of this region may occur when the transporters are in their normal cellular context in *C. elegans*.

### Functional activity of FGT1 in *C. elegans*

**Figure 3:**
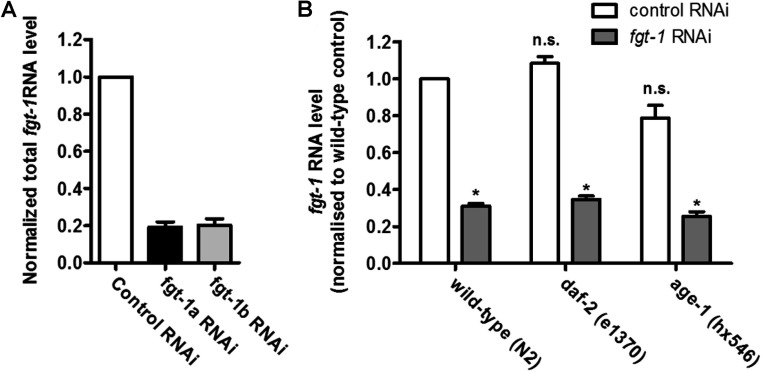
Knockdown of *fgt-1a* and *fgt-1b* in wild-type and signalling mutant *C. elegans* strains (**A**) *C. elegans* wild-type strain (N2) L1 larvae were seeded on to NGM plates seeded with *fgt-1a* or *fgt-1b* RNAi bacteria (with or without 1 mM IPTG to induce knockdown). The young adult worms were transferred on to NGM plates (50 μM FUDR, with or without 1 mM IPTG) seeded with *fgt-1a* or *fgt-1b* RNAi bacteria. The worms were maintained on the plates without exhaustion of bacteria food source for 10 days. Total RNAs of each sample were extracted and the extent of knockdown of the total *fgt-1* mRNA was determined by qPCR using primers to a region present in both transcripts. Results are means±S.E.M. for three separate experiments. (**B**) *C. elegans* L1 larvae of wild-type strain (N2) or strains mutated in the DAF-2 receptor [*daf-2* (e1370)], or the PI3K [*age-1* (hx546)] were seeded on to NGM plates seeded with *fgt-1a* and *fgt-1b* RNAi bacteria mixture (1:1 ratio) (with or without 1 mM IPTG to induce knockdown). The young adult worms were transferred to NGM plates (50 μM FUDR, with or without 1 mM IPTG) seeded with *fgt-1a* and *fgt-1b* RNAi bacteria mixture (1:1 ratio). The worms were maintained on the plates without exhaustion of bacteria food source for 10 days. Total RNA of each sample were extracted and the extent of knockdown of the total *fgt-1* mRNA was determined by qPCR using primers to a region present in both transcripts. Results are means±S.E.M. for three separate experiments. *P<0.05 compared with the uninduced control. mRNA levels in mutant strains *daf-2* and *age-1* were not significantly different (n.s.) compared with the wild-type strain (*P*=0.072 and 0.075 respectively).

**Figure 4:**
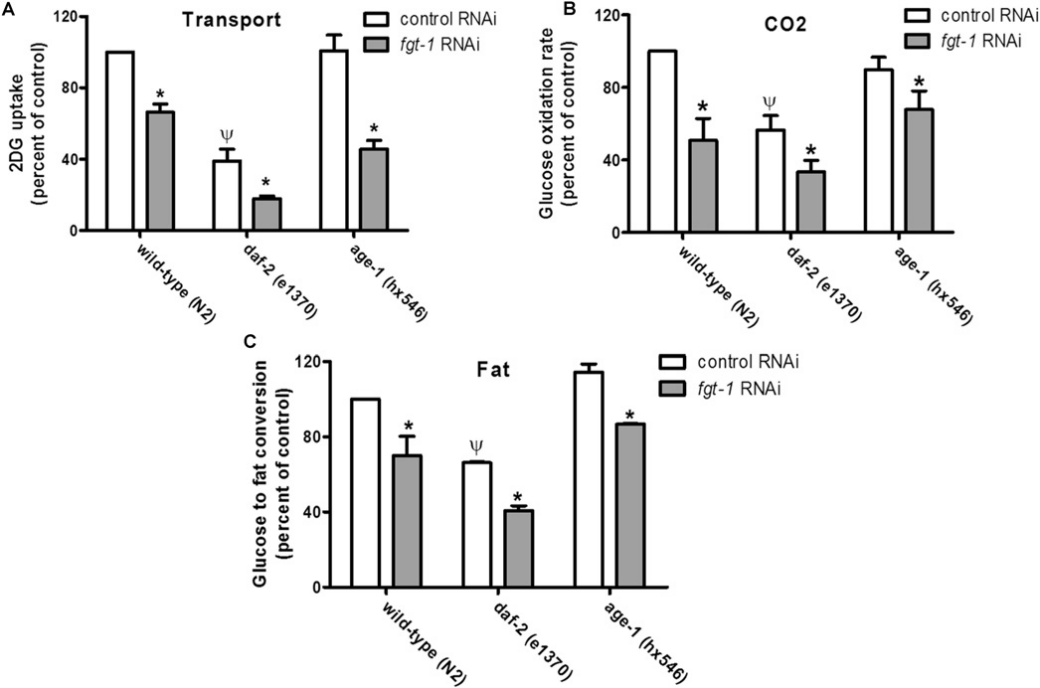
Knockdown of *fgt-1* influences whole-body glucose uptake and metabolism in *C. elegans* (**A**) Whole-body 2DG uptake in the indicated *C. elegans* strains was determined. *P<0.05 compared with the control uninduced RNAi. Ψ*P*<0.05 compared with the wild-type *C. elegans* strain fed with control uninduced RNAi bacteria. (**B**) Whole-body glucose oxidation in the indicated *C. elegans* strains was determined from the conversion of medium 500 μM [^14^C]glucose into ^14^CO_2_ which was trapped in KOH. *P<0.05 compared with the control RNAi. Ψ*P*<0.05 compared with the wild-type *C. elegans* strain fed with control uninduced RNAi bacteria. (**C**) Whole-body glucose into fat conversion in the indicated *C. elegans* strains was determined from the ^14^C fat content extracted from worm lysates with heptane. *P<0.05 compared with the control RNAi. Ψ*P*<0.05 compared with the wild-type *C. elegans* strain fed with control RNAi bacteria. Results are means±S.E.M. for three separate experiments.

To determine whether FGT-1 is important in the uptake of glucose from the medium into the intact animal we used an RNAi approach. Worms were fed *Escherichia coli* cells expressing either the *fgt-1a* or *fgt-1b* RNAi constructs alone (Figure 3A) or as a 1:1 mixture of *fgt-1a* and *fgt-1b* RNAi constructs (Figure 3B). The single as well as the combined RNAi construct knockdowns led to 70–80% reductions in the combined *fgt-1* transcripts. These data confirmed that knockdown of both *fgt-1* transcripts by a single RNAi corresponding to any region of the *fgt-1* gene leads to silencing of the whole gene and both splice variants. The observed level of reduction in the *fgt-1* transcripts was sufficient to produce significant reductions in 2DG uptake (Figure 4A), glucose oxidation (Figure 4B) and glucose conversion into triglyceride (Figure 4C) in intact worms.

It has recently been suggested that an unidentified glucose transport may be regulated downstream of the DAF-2 receptor in *C. elegans* [7]. The results of the present study suggest that the FGT-1 transporters may fulfil this role. To examine the possibility that alteration in the genetic background of the worms might alter the effects of FGT-1 reduction in glucose oxidation we examined this process in *daf-2* [24] and *age-1* [15] loss-of-function mutant worms. It was found that the *daf-2* mutants had significantly reduced levels of glucose uptake and oxidation and conversion into triglyceride (Figures 4B and 4C). This is consistent with data on 2DG uptake in worms fed a *daf-2*-knockdown construct [7]. This suggests that glucose uptake and metabolism is dependent on the DAF-2 receptor. On the wild-type, *daf-2* and *age-1* backgrounds, FGT-1 reduction resulted in significant further inhibition of glucose uptake and oxidation (Figure 4).

### Dependence of *C. elegans* lifespan on FGT-1 activity

**Figure 5:**
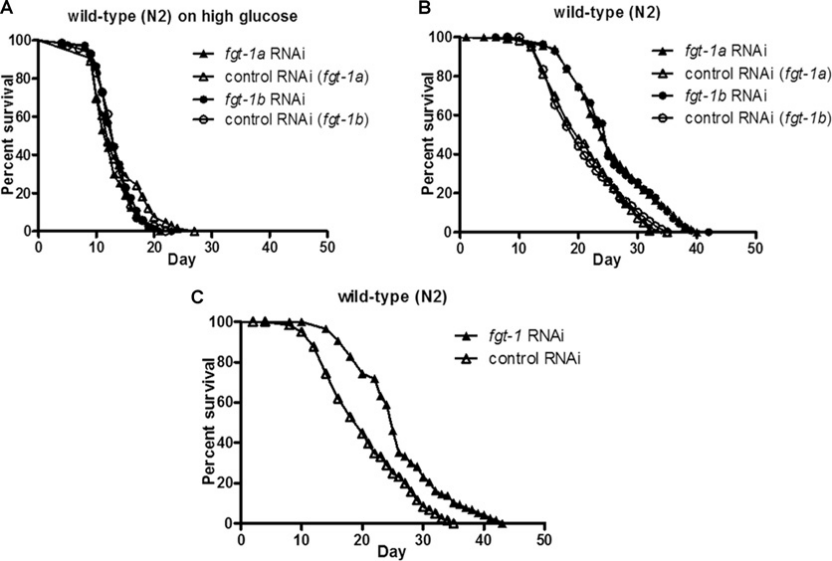
*fgt-1* knockdown extends lifespan in *C. elegans* (**A**) Determination of lifespan for wild-type (N2) worms exposed to *fgt-1a* RNAi (▲) and *fgt-1b* RNAi (●) with high (20 mM) glucose concentration medium. ∆ and ○, control uninduced RNAi. (**B**) Determination of lifespan for wild-type (N2) worms exposed to *fgt-1a* RNAi (▲) and *fgt-1b* RNAi (●) together with NGM. ∆ and ○, control uninduced RNAi. (**C**) Determination of lifespan for wild-type (N2) worms exposed to a *fgt-1a* and *fgt-1b* RNAi mixture (1:1 ratio) together with NGM. ▲, *fgt-1* RNAi; ∆, control uninduced RNAi.

We next examined whether the FGT-1 transporters might influence longevity in *C. elegans*. Such a lifespan-extending effect might be anticipated from the known longevity effects of nutrient restriction [6,25] and reduced DAF-2 signalling [15,24], both of which may mediate a lowering of glucose uptake. We have confirmed [8] (Figures 5A and 5B) that high-glucose levels (20 mM) reduce the lifespan of worms and this effect is not reduced by *fgt-1* mRNA knockdown. The lack of effect of 70–75% knockdown of *fgt-1* transcripts in worms taking up high levels of glucose may occur because knockdown on this background is insufficient to reduce glucose-derived energy metabolites to a level that influences lifespan; however, knockdown of *fgt-1* leads to an increase in lifespan on medium nominally free of glucose, where any available sugar is derived from bacteria or worm metabolism of ingested bacteria. We obtain the same lifespan-extending phenotype in nominally glucose-free medium using either RNAi alone (Figure 5B) or by a combination of *fgt-1a* and *fgt-1b* RNAi (Figure 5C). The maximum lifespan with the *fgt-1* knockdown is extended from 35 to 42 days, whereas the mean lifespan is extended from 20 to 25 days. Thus the extension to lifespan is 20–25% (*P*<0.0001; Table 2). The lifespan-extension effect of *fgt-1* knockdown is equivalent to that in *daf-2* and *age-1* worms.

**Table 2:** Statistical analysis of lifespan extension in *C. elegans* Statistical analysis was performed with Kaplan–Meier method. Data are the combined values for two independent experiments.

Strain (RNAi)	Maximum lifespan (days)	Mean lifespan (days)	*P* value (compared with the control)	Number of nematodes (censored)
N2 (*fgt-1a* RNAi, high glucose)	21	12	0.0603	64 (0)
N2 (control RNAi (*fgt-1a*), high glucose)	27	12		66 (0)
N2 (*fgt-1b* RNAi, high glucose)	23	13	0.8005	74 (0)
N2 (control RNAi (*fgt-1b*), high glucose)	22	13		70 (0)
N2 (*fgt-1* RNAi)	43.0	25.0	<0.0001	122 (3)
N2 (control RNAi)	35.0	20.0		124 (5)
N2 (*fgt-1a* RNAi)	40.0	24.0	<0.0001	141 (1)
N2 (control RNAi (*fgt-1a*))	35.0	20.0		124 (6)
N2 (*fgt-1b* RNAi)	42.0	25.0	<0.0001	116 (4)
N2 (control RNAi (*fgt-1b*))	35.0	20.0		131 (3)
*daf-2*(e1370) (*fgt-1a* RNAi)	46.0	29.5	0.0001	128 (3)
*daf-2*(e1370) (*fgt-1a* RNAi control)	42.0	27.0		131 (4)
*daf-2*(e1370) (*fgt-1b* RNAi)	47.0	28.0	0.0232	128 (5)
*daf-2*(e1370) (*fgt-1b* RNAi control)	42.0	26.0		150 (0)
*daf-2*(e1370) (*fgt-1* RNAi)	47.0	29.0	0.0035	123 (4)
*daf-2*(e1370) (control RNAi)	41.0	26.0		126 (0)
*age-1*(hx546) (*fgt-1a* RNAi)	43.0	26.0	0.0009	108 (0)
*age-1*(hx546) (*fgt-1a* RNAi control)	41.0	25.0		115 (1)
*age-1*(hx546) (*fgt-1b* RNAi)	42.0	26.0	0.0148	126 (1)
*age-1*(hx546) (*fgt-1b* RNAi control)	44.0	25.0		110 (5)
*age-1*(hx546) (*fgt-1* RNAi)	44.0	27.0	0.0626	116 (0)
*age-1*(hx546) (control RNAi)	41.0	26.0		121 (4)

**Figure 6:**
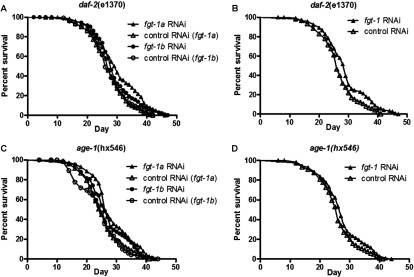
The lifespan-extending effects of *fgt-1* knockdown are reduced on the *daf-2* background, whereas there is no significant lifespan extension on the *age-1* background (**A**) Determination of lifespan for *daf-2* (*e1370*) mutant worms exposed to *fgt-1a* RNAi (▲) and *fgt-1b* RNAi (●) with NGM. ∆ and ○, control uninduced RNAi. (**B**) Determination of lifespan for *daf-2* (*e1370*) mutant worms exposed to a *fgt-1a* and *fgt-1b* RNAi mixture (1:1 ratio) together with NGM. ▲, *fgt-1* RNAi; ∆, control RNAi. (**C**) Determination of lifespan for *age-1* (*hx546*) mutant worms exposed to *fgt-1a* RNAi (▲) and *fgt-1b* RNAi (●) together with NGM. ∆ and ○, control uninduced RNAi. (**D**) Determination of lifespan for *age-1* (*hx546*) mutant worms exposed to a *fgt-1a* and *fgt-1b* RNAi mixture (1:1 ratio) together with NGM. ▲, *fgt-1* RNAi; ∆, control uninduced RNAi.

The lifespan-extending effects of *fgt-1* knockdown are reduced from 7 to 2.5 days on the *daf-2* background (Figures 6A and 6B), whereas there is no significant lifespan extension on the *age-1* background (Figures 6C and 6D, and Table 2).

## DISCUSSION

### Glucose transporter identification

Bioinformatics approaches have identified a large number of *C. elegans* sequences as belonging to the sugar transporter family; however, we show in the present study that this approach in *C. elegans* leads to misassignments, as only FGT-1 isoforms (A and B) are functional. While the present study was in progress [26], Kitaoka et al. [27] reported similar findings on the transport activity of the FGT-1A isoform. Alignment of all the *C. elegans* putative transporter sequences with GLUT1 (Figure 1 and Supplementary Figure S1) suggests that many of these proteins have charged residues lying centrally within the TM segments of the C-terminal half of the protein, and particularly in the TM7 section which is thought to be involved in substrate recognition (Supplementary Table S3 at http://www.biochemj.org/bj/456/bj4560219add.htm). However, the *bona fide* mammalian GLUT transporters in the Class1 subfamily do not have charged residues in these regions. Therefore the lack of charged residues in TM segments could be considered an important signature characteristic for facilitative glucose transporters in general.

The recent crystallization of XylE, the bacterial homologue of the Class1 GLUT transporters, and the modelling of the GLUT1 structure has highlighted the importance of the TM7 region in substrate binding deep within an exofacially directed cleft in the transporter [20]. Invariant and non-charged residues in this region are therefore probably essential for glucose recognition. The critically conserved regions necessary for sugar substrate binding around TM7 [28–31] are clearly present in the FGT-1 *C. elegans* transporters (Figure 1). In particular the critical QQLSGIN and YST sequences [22] that mutagenesis studies on GLUT1 have shown to be essential are present in these *C. elegans* sequences. The FGT-1 proteins are described in WormBase as the *C. elegans* GLUT3 homologues (43% identity), but also have significant identity with GLUT1 (37%) and GLUT4 (35%) (Table 1). The FGT-1 proteins are therefore likely to be functionally equivalent to the regulated facilitative glucose transporters in mammals.

Even within the mammalian GLUT family of proteins some misassignment of glucose as the main substrate of the putative transporters appears to have occurred. The GLUT family of proteins is now divided into three classes based on phylogenetic and bioinformatics approaches. The Class1 GLUTs are functionally well characterized as glucose transporters. This class includes the GLUT1 found in most mammalian cells, GLUT2 found mainly in the liver and pancreatic β-cells, GLUT3 found mainly in the brain, and GLUT4 found mainly in insulin-responsive adipose, heart and skeletal muscle tissue. It is these transporters that are primarily responsible for maintaining normal glucose homoeostasis and endocrine-regulated glucose uptake. For the Class2 and Class3 GLUTs the substrate preferences and specificities of the transporters are unclear and there are emerging examples of misassignment of these proteins as glucose transporters. Functionally well-characterized transporters include GLUT9 (in Class2) which is now known to have a strong substrate specificity for urate [32], whereas HMIT (in Class3) is a H^+^ symporter for myo-inositol [33].

These comparisons suggest that many of the *C. elegans* GLUT homologues may have alternative facilitative transport substrates or may exhibit ion-coupled active transport. Future studies could be aimed at identifying the preferred substrates of these proteins.

### Regulation of glucose transport and metabolism in *C. elegans*

The results of the present study lead us to conclude that FGT-1 is the major transporter responsible for glucose uptake in *C. elegans* as a 70% reduction in the mRNA for this protein leads to a highly significant reduction in glucose transport. FGT-1 is therefore likely to be responsible for maintaining basal non-stimulated levels of glucose transport. We found in the present study that glucose transport activity and associated glucose metabolism are reduced in worms with an inactivating mutation in *daf-2*. This suggests that FGT-1s are also responsible for stimulated uptake in *C. elegans*. These data are consistent with the findings from Zarse et al. [7] who have reported that transient knockdown of DAF-2 leads to reduction in glucose transport.

We have confirmed that when worms are incubated with high (20 mM) glucose there is a very marked reduction in normal lifespan [8]. We report in the present study that lifespan can be extended beyond the normal level (and while the worms consume only bacteria from the medium) by knockdown of *fgt-1*. The lifespan-extending effects of *fgt-1* knockdown are reduced from 7 to 2.5 days on the *daf-2* background, whereas there is no significant lifespan extension on the *age-1* background. In studies in which calorie restriction and *daf-2* mutation have been combined the lifespan-extending effects have been reported to be additive, suggesting independence of these effects [34] or non-additive, suggesting that they are on a common pathway [7,35]. The results of the present study suggest that the major part of glucose uptake in *C. elegans* may be controlled by signalling, as reduction in FGT-1-mediated glucose transport increases lifespan to a level that is similar to a reduction in DAF-2 signalling. The involvement of AGE-1/PI3K in the signalling route between AGE-1 and FGT-1 is less clear and non-PI3K routes from AGE-1 signalling to glucose transport may occur. Divergence in signalling to glucose transport also occurs in mammals [2,36,37].

### The insulin-sensitivity and healthy-aging dichotomy

Reduced signalling from insulin and insulin-like peptides leads to increased lifespan in all species [4]. In mammals this effect occurs when reduced signalling is confined to specific tissues. Mice live longer when the insulin receptor is knocked out, specifically in fat tissue [11]. Likewise brain-specific knockout of IRS2 (insulin receptor substrate 2) in mice extends lifespan [38]. By contrast, a common strategy for combating the insulin resistance that is associated with Type 2 diabetes is treatment with insulin sensitizers which increase tissue glucose uptake [2,39,40]. Although there are clear benefits of these strategies for lowering blood glucose and reducing associated cardiovascular damage, the treatments seem somewhat at odds with the lifespan-extending effects of reduced insulin signalling [6].

In mammalian insulin-sensitive tissues, the GLUT4 glucose transporter is highly sequestered in a specialized intracellular compartment. This system may be evolutionarily conserved through the conservation of thrifty genes, such as the RabGAPs (Rab GTPase-activating proteins), that sequester the glucose transporters in intracellular compartments of hormonally controlled tissues so that blood glucose is spared for the brain [41]. The present study on the glucose-transport dependence of lifespan in *C. elegans* leads us to suggest that healthy aging strategies could be more directed towards sequestering glucose-transport activity and maintaining low-basal glucose-transport rates than towards increasing insulin sensitivity. Such a strategy would have to be associated with measures to reduce unnecessary nutrient intake in humans over extended periods [7].

In *C. elegans* and in mice reduction in glucose uptake have been shown to lead to increased AMPK activity, increased mitochondrial activity, increased stress response to ROS and beneficial effects on life expectancy [6,11]. Low-calorie diets are associated with improved health outcomes in mammals and humans [42–46]; however, directly attributing effects of nutrient restriction to changes in glucose uptake is more problematic in studies on mammals than in simple organisms, as a wide range of tissue-specific responses are known to contribute to maintaining normal glycaemia [47]. Nutrient restriction can greatly lower glycaemia of hyperglycaemic animals with more moderate effects in normoglycaemic animals [48].

Polyphenolic compounds that extend lifespan in *C. elegans* may act on a range of targets including sirtuins and ROS [49,50]; however, controversies exist concerning these effects [5,51]. A range of polyphenolic compounds, including phloretin that we have used here as a transport blocker, are known to interact strongly with GLUT1 in human erythrocytes [52] and thus may target FGT-1. Further studies could therefore usefully determine whether some of the longevity effects of polyphenolic compounds in *C. elegans* may occur through alterations in glucose uptake.

The results of the present study suggests that the FGT-1A and -B are the major, and maybe the only, GLUT-like proteins in *C. elegans* that are functional glucose transporters. The discovery of a lifespan extending role for FGT-1 suggests that it may be a therapeutic target for healthy aging. The lifespan-extending effects of DAF-2 and AGE-1 are known to be complex and involve many distinct targets [10,11]. However, the discovery that FGT-1 in *C. elegans* is a paralogue of the mammalian GLUT transporters, and may constitute a common axis for hormonal and nutritional control of energy metabolism, suggests that further studies on this system in the relatively simple *C. elegans* model of aging will have direct bearing on the more complex problem of investigation of healthy aging in humans.

## Online data

Supplementary data
