# Supplementary material for: FGT-1 is the major glucose transporter in C. elegans and is central to aging pathways
Source: Biochem J. 2013 Nov 8;456(Pt 2):219–29. doi: 10.1042/BJ20131101 (PMC3898116; doi:10.1042/BJ20131101)
Supplement: Supplementary data [file bj4560219add.pdf]

BJ Metabolism

Biochem. J. (2013) **456**, 219–229 (Printed in Great Britain) doi:10.1042/BJ20131101

online data

SUPPLEMENTARY ONLINE DATA

FGT-1 is the major glucose transporter in *C. elegans* and is central to aging pathways

Ying FENG*[1], Barnabas G. WILLIAMS†, Françoise KOUMANOV*, Adrian J. WOLSTENHOLME† and Geoffrey D. HOLMAN*[2]

*Department of Biology and Biochemistry, University of Bath, Bath, BA2 7AY, U.K., and †Department of Infectious Diseases, University of Georgia, Athens, GA 30602, U.S.A.

Table S1 Primers used for cloning the putative glucose transporter genes from a *C.elegans* cDNA library

Gene Name	Gene size (bp)	Primer name	Primer sequence (5′→3′)
fgt-1a	1479	H17aFOR	AAATGGGTGTCAACGACC
		H17aREV	GCATTACTTCCTCTTCTCG
fgt-1b	1533	H17bFOR	ATGTCGGAAAAATCAAGAAGTG
		H17bREV	TTACTTCCTCTTCTCGAATTCG
		H17int	GGCCACTGATCTTTGCC
R09B5.11	1551	R09FOR	ATGAACGCC GTTGTTGCC
		R09REV	CCGACTATGAACATATCCG
		R09intFOR	GGTGCCAA TGTTTCTTACCG
		R09intREV	CGGTAAGAAACATTGGCACC
C35A11.4	1548	C35FOR	ATGGTGGAAGCACCCAGTTTTCGG
		C35REV	CTAGAGTCGATAAGATAAGG
		C35intFOR	CTGTGAGATTGATGAAGCC
		C35intREV	GGCTTCATCAATCTCACAG
F14E5.1	1419	F14FOR	ATGTCAAATAGATTGTGGCC
		F14REV	TCATTTTTTTTTAAGTTCATTC
		F14intFOR	GGAGATGGTACAGATGC
		F14intREV	GCATCTGTACCATCTCC
Y39E4B.5	1518	Y39FOR	ATGCGGTGGCAAACGATTCG
		Y39REV	TTAGTAAATTCGAAGATGTCC
		Y39intFOR	CATGAGCGAATATCAAGCC
		Y39intREV	GGCTTGATATTCGCTCATG
F48E3.2	1467	F48FOR	CCAGTGACAACATGCAAAAACTCAATTGG
		F48REV	GGCTGTCATTATAATTTATTCATTCC
		F48intFOR	CGTCGTGGGTTTTTGTTGGG
		F48intREV	CCCAACAAAAACCCACGACG
K09C4.5	1575	K4.5FOR	CCATTAGCAATCATGTTATTCAATGCGCC
		K4.5REV	GGCAATGAAGCAAGCAAACATCGCAGG
		K4.5intFOR	GGAGAAGAATTTGACACAGG
		K4.5intREV	CCTGTGTCAAATTCTTCTCC
T08B1.1	1776	T08FOR	ATGTCAGAATTTGAAGAAG
		T08REV	CAGTCTTCCTTCACATTTGG
		T08intFOR	CGTCCTGACCTACGCTATGG
		T08intREV	CCATAGCGTAGGTCAGGACG

Table S2 Insertion sites for the exofacial HA tag in putative *C. elegans* transporters

The influenza HA epitope (peptide sequence, IDYPYDVPDYAE) was introduced using the sense oligonucleotide: 5′-TGAGATCGATTATCCTTATGATGTTCCTGATTATGC-3′ and the antisense oligonucleotide: 5′-TCAGCATAATCAGGAACATCATAAGGATAATCGATC-3′. A specific restriction enzyme site (Bsu36I) was created by site-directed mutagenesis (at the position indicated in the Table) so that the sticky ends could be produced after the digestion. Subsequently the HA tag was ligated into the sequence with correct orientation. The consequent mutated amino acids for each protein are listed.

Gene name	Position of the HA insertion	Mutated amino acid
H17B01.1a	Thr^{57}–Glu^{58}	–
H17B01.1b	Pro^{70}–Gly^{71}	–
R09B5.11	Pro^{71}–Gly^{72}	–
C35A11.4	Met^{42}–Asn^{43}	Met^{42}→Pro, Asn^{43}→Asp
F14E5.1	Thr^{55}–Glu^{56}	–
Y39E4B.5	Leu^{45}–Arg^{46}	Arg^{46}→Ala, Gln^{47} → Glu, Pro^{48}→Ala
F48E3.2	Asn^{42}–Glu^{43}	–
K09C4.5	Pro^{51}–Thr^{52}	Thr^{52}→Ala
K09C4.1	Thr^{54}–Glu^{55}	Thr^{54}→Glu
T08B1.1	Asp^{76}–Trp^{77}	Asp^{76}→Glu, Trp^{77}→Gly

[1] Present address: Department of Histoembryology and Neurobiology, College of Basic and Forensic Medicine, Sichuan University, Chengdu, Sichuan, 610041, People's Republic of China.

[2] To whom correspondence should be addressed (email g.d.holman@bath.ac.uk).

The gene and protein names for *fgt-1a* and *fgt-1b* and FGT-1A and -1B have been deposited in WormBase (http://www.wormbase.org) under accession numbers NM_061580.4 and NM_061581.1 respectively.

© 2013 The Author(s)

The author(s) has paid for this article to be freely available under the terms of the Creative Commons Attribution Licence (CC-BY) (http://creativecommons.org/licenses/by/3.0/) which permits unrestricted use, distribution and reproduction in any medium, provided the original work is properly cited.

Biochemical Journal www.biochemj.org

Table S3 Positions of charged residues in putative TM segments

Charged residues in putative TM segments were identified following sequence alignment (Figure S1). The Table includes charged residues within the central eight residues in the proposed TM regions, but excludes charged residues in the first or last three residues of the proposed helices. The membrane topology of each putative transporter was set up to maximize alignment with the hGLUT1 TM regions and at the same time taking account of the membrane topology predicted by the SOSUI program.

	Putative charged residue positions in TM segments											
Strain	TM1	TM2	TM3	TM4	TM5	TM6	TM7	TM8	TM9	TM10	TM11	TM12
FGT-1A		Asp^{78}		Arg^{142}				Asp^{346}				
FGT-1B		Asp^{96}		Arg^{160}				Asp^{364}				
R09B5.11	Asp^{61}	Asp^{97}		Arg^{161}	Lys^{190}			Lys^{354}, Asp^{366}				
C35A11.2				Arg^{124}		Glu^{191}	Asp^{297}	Asp^{310}, Asp^{328}, Arg^{329}			Arg^{416}	
F14E5.1		Asp^{60}		Arg^{124}	Glu^{159}, Asp^{175}			Lys^{309}	Lys^{357}, Arg^{359}			
Y39E4B.5				Arg^{122}, Asp^{133}	Glu^{157}, Lys^{175}	Arg^{182}		Asp^{324}, Arg^{325}			Asp^{418}	
F48E3.2		Glu^{57}		Arg^{121}	Glu^{156}		Arg^{266}, Asp^{290}	Lys^{304}				
K09C4.5	Asp^{20}		Arg^{97}	Arg^{134}	Asp^{162}		Asp^{273}	Asp^{319}, Arg^{311}	Lys^{338}	Glu^{365}, Glu^{369}		
K09C4.1	Asp^{20}	Glu^{70}	Arg^{97}		Lys^{166}	Glu^{191}	Asp^{281}, Asp^{292}	Asp^{327}, Lys^{319}	Lys^{342}, Lys^{345}, Glu^{354}	Glu^{373}, Arg^{388}		

© 2013 The Author(s)
The author(s) has paid for this article to be freely available under the terms of the Creative Commons Attribution Licence (CC-BY) (http://creativecommons.org/licenses/by/3.0/) which permits unrestricted use, distribution and reproduction in any medium, provided the original work is properly cited.

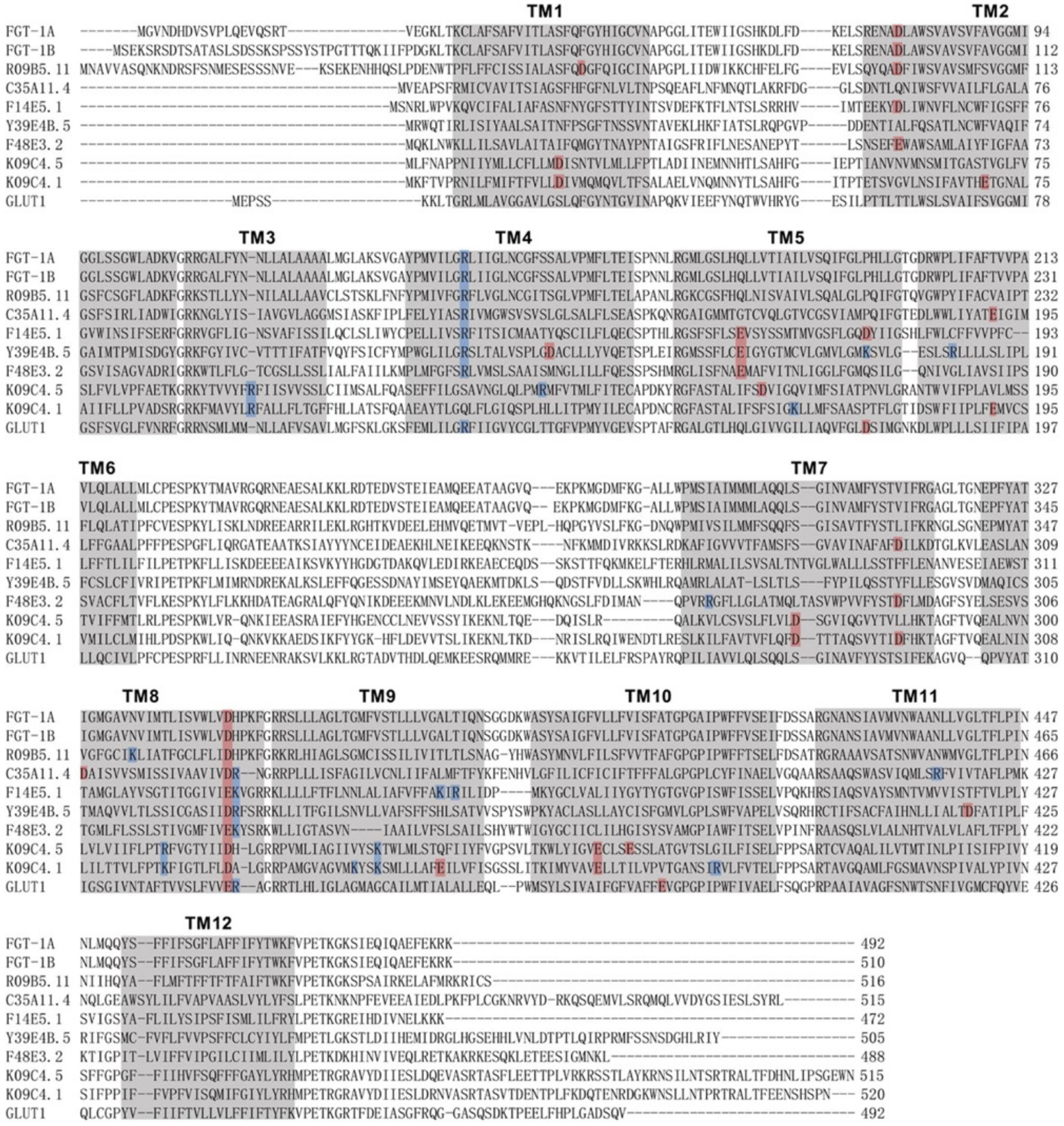

Figure S1 Alignments of GLUT1 with putative transporters cloned from *C.elegans*

Many of the putative *C. elegans* transporters have charged residues (blue and red shading) in regions that correspond to the mammalian GLUT protein TM regions. We propose that this property may be associated with poor ability to facilitate glucose transport and that the property is likely to be more associated with alternative transported substrates.

Received 16 August 2013/10 September 2013; accepted 11 September 2013
Published as BJ Immediate Publication 11 September 2013, doi:10.1042/BJ20131101

© 2013 The Author(s)
The author(s) has paid for this article to be freely available under the terms of the Creative Commons Attribution Licence (CC-BY) (http://creativecommons.org/licenses/by/3.0/) which permits unrestricted use, distribution and reproduction in any medium, provided the original work is properly cited.
